# Improvement of osseointegration of Ti–6Al–4V ELI alloy orthodontic mini-screws through anodization, cyclic pre-calcification, and heat treatments

**DOI:** 10.1186/s40510-022-00405-8

**Published:** 2022-04-04

**Authors:** Changkyun Im, Je-Hyeok Park, Young-Mi Jeon, Jong-Ghee Kim, Yong-Seok Jang, Min-Ho Lee, Woo-Yong Jeon, Jun-Min Kim, Tae-Sung Bae

**Affiliations:** 1 Dental Research Institute, Seoul National University School of Dentistry, Seoul, Republic of Korea; 2 Department of Orthodontics, School of Dentistry, Jeonbuk National University, Jeonju, Republic of Korea; 3 BK21 Plus Program, Department of Dental Biomaterials, Institute of Biodegradable Materials, School of Dentistry, Jeonbuk National University, Jeonju, Republic of Korea; 4 Research Institute of Clinical Medicine of Chonbuk National University, Biomedical Research Institute of Chonbuk National University Hospital, Jeonju, Republic of Korea; 5 Department of Dental Technology, Gwangyang Health College, Gwangyang, Republic of Korea; 6 Department of Electronics and Information Engineering, Hansung University, Seoul, 02876 Republic of Korea

**Keywords:** Ti–6Al–4V ELI alloy, Orthodontic mini-screw, Anodization, Cyclic pre-calcification, Osseointegration

## Abstract

**Background:**

Mini-screws are widely used as temporary anchorages in orthodontic treatment, but have the disadvantage of showing a high failure rate of about 10%. Therefore, orthodontic mini-screws should have high biocompatibility and retention. Previous studies have demonstrated that the retention of mini-screws can be improved by imparting bioactivity to the surface. The method for imparting bioactivity proposed in this paper is to sequentially perform anodization, periodic pre-calcification, and heat treatments with a Ti–6Al–4V ELI alloy mini-screw.

**Materials and methods:**

A TiO_2_ nanotube-structured layer was formed on the surface of the Ti–6Al–4V ELI alloy mini-screw through anodization in which a voltage of 20 V was applied to a glycerol solution containing 20 wt% H_2_O and 1.4 wt% NH_4_F for 60 min. Fine granular calcium phosphate precipitates of HA and octacalcium phosphate were generated as clusters on the surface through the cyclic pre-calcification and heat treatments. The cyclic pre-calcification treatment is a process of immersion in a 0.05 M NaH_2_PO_4_ solution and a saturated Ca(OH)_2_ solution at 90 °C for 1 min each.

**Results:**

It was confirmed that the densely structured protrusions were precipitated, and Ca and P concentrations, which bind and concentrate endogenous bone morphogenetic proteins, increased on the surface after simulated body fluid (SBF) immersion test. In addition, the removal torque of the mini-screw fixed into rabbit tibias for 4 weeks was measured to be 8.70 ± 2.60 N cm.

**Conclusions:**

A noteworthy point in this paper is that the Ca and P concentrations, which provide a scaffold suitable for endogenous bone formation, further increased over time after SBF immersion of the APH group specimens. The other point is that our mini-screws have a significantly higher removal torque compared to untreated mini-screws. These results represent that the mini-screw proposed in this paper can be used as a mini-screw for orthodontics.

**Supplementary Information:**

The online version contains supplementary material available at 10.1186/s40510-022-00405-8.

## Introduction

In orthodontic treatment, functionally stable and strong anchorage is the most important factor to move teeth to the ideal position. Orthodontic mini-screw is a temporary skeletal anchorage device, which is a useful clinical tool that can move the teeth to the target position while minimizing patient cooperation.

The mini-screw must be functionally stable in the alveolar bone during the procedure to move the tooth to the ideal position. However, if it has identifiable mobility or becomes loose, it cannot provide ideal tooth movement, increasing treatment time and leading to poor treatment [1, 2]. Therefore, the orthodontic mini-screw must have biocompatibility, excellent mechanical properties, good osseointegration, high corrosion resistance, and excellent wear resistance [3].

Titanium (Ti) is one of the few biomaterials that meet these requirements. In the field of orthodontics, titanium alloys with superior strength than commercially pure titanium (cp Ti) having insufficient strength are mainly used because of the characteristics that mini-screws must have excellent mechanical properties [4]. Among titanium alloys, Ti–6Al–4V alloy, which has excellent mechanical properties, chemical stability, and biocompatibility, is used as an implant material [3, 5, 6]. However, it is known that the orthodontic mini-screw made of Ti–6Al–4V alloy also lacks mechanical retention with the teeth, resulting in a failure rate of about 10% within 2 months [7, 8].

One of the proven strategies to increase the success rate is to accelerate early osseointegration to achieve rapid stability [9, 10]. Accordingly, several surface treatment methods have been proposed to improve early osseointegration. As an example, there is a method of coating hydroxyapatite which is a component of bone to modify the surface of a titanium or titanium alloy implant to have bioactivity [11, 12]. However, although this method has been in the spotlight as a surface treatment method that combines the mechanical properties of titanium and osteoconduction of hydroxyapatite(HA), there is a problem that the coating layer is easily peeled off because it is difficult to obtain a strong bonding force between the coating layer and titanium [13, 14]. In recent studies, as it is known that an appropriately rough surface plays an important role in increasing the osseointegration rate and stability of an implant compared to a smooth surface [15–17], attention is focused on the method of structuring the surface. In particular, as it is known that the nanostructured surface provides a wider contact area than the micron-structured surface to promote the adhesion and proliferation of osteoblasts [18], various methods of forming nanostructures on the surface are being actively studied. Among them, a method of forming a nanotube-structured titanium dioxide (TiO_2_) layer by anodizing in an electrolyte solution containing fluorine ions, and then forming a nano-thick bioactive coating layer by pre-calcification treatment is attracting attention [13, 19–21]. It has been reported that the formation of a nanotube-structured TiO_2_ layer increases the surface area by about 46 times compared to a smooth surface [22].

Pre-calcification treatment is one of the ways to improve early osseointegration. The pre-calcification treatment is a surface treatment method that induces an acid–base reaction between the TiO_2_ layer and these ions by treating titanium in a solution containing phosphate and calcium ions, which are the main components of HA [21]. Kodama et al. [23] announced that HA precipitation was promoted when a nanotube-structured TiO_2_ layer was formed on the surface of pure titanium or titanium alloy, and circulated 20 times each in 0.02 M NH_4_H_2_PO_4_ and saturated Ca(OH)_2_ solutions, and then immersed in the simulated body fluid (SBF). Nguyen et al. [19, 20] reported that the surface of the Ti-6Al-7Nb alloy has a marked increase in bioactivity by treating in a glycerol electrolyte solution containing 20 wt% water (H_2_O) and 1 wt% ammonium fluoride (NH_4_F) to form a nanotube-structured layer, and then circulated 20 times each in 0.05 M NaH_2_PO_4_ and saturated Ca(OH)_2_ solutions.

Therefore, this study investigated whether the Ti–6Al–4V ELI alloy mini-screw applied with cyclic pre-calcification treatment was suitable for use as a temporary anchorage device through SBF immersion, cytotoxicity, and removal torque tests.

## Material and methods

In order to investigate the suitability of the Ti–6Al–4V ELI alloy mini-screw with cyclic pre-calcification applied as a temporary anchorage device for orthodontic treatment, this study was carried out by dividing into (1) untreated (UT), (2) anodized and heat-treated (AH), 3) anodized, pre-calcified, and heat-treated (APH) groups.

### Specimen preparation

In this study, commercially available Ti–6Al–4V ELI alloy plates (Kobe Steel Ltd, Japan) cut into a size of 20 mm × 10 mm × 1 mm were used as plate specimens. The plate specimens were sequentially polished with Silicon Carbide (SiC) abrasive paper of grit size from 220 to 1000, ultrasonically cleaned in alcohol and acetone solution for 5 min, respectively, and dried for 24 h before use. For use in the animal implantation test, mini-screws with a diameter of 1.4 mm and a length of 3.3 mm were manufactured by computer numerical control (CNC; Cincom L20, Citizen Machinery Co, Ltd, Miyota, Japan) machining from a Ti–6Al–4V ELI alloy rod (Fort Wayne Metals Research Products Co, USA) with a diameter of 4 mm and a length of 3 m.

### Anodization treatment

As a pre-anodization treatment, pickling treatment was performed to remove the existing oxide layer present on the surface layer of specimens and mini-screws by immersing in a 12:7:81 solution mixed with nitric acid (HNO_3_), hydrofluoric acid (HF), and H_2_O for 10 s. Anodization treatment was performed by connecting the specimen and the platinum plate to the anode and cathode of a DC electrostatic source, respectively, and putting them in a glycerol electrolyte solution containing 20 wt% H_2_O and 1.4 wt% NH_4_F. The nanotube-structured TiO_2_ layer was formed by holding for 60 min under the conditions of a voltage of 20 V and a current density of 20 mA/cm^2^. The surface on which the nanotube-structured layer was formed was ultrasonically cleaned with distilled water for 1 min, stored in a dryer at 50° C for at least 24 h, and then used in following tests.

### Cyclic pre-calcification treatment

Before the cyclic pre-calcification treatment, the specimen was immersed in 0.5 vol% silicate solution for 5 min and then dried at 100° C. for 1 h. To induce the precipitation of calcium phosphate on the specimen, cyclic pre-calcification treatment was performed 20 times by immersing in each of a 0.05 M NaH_2_PO_4_ solution at 90 °C and a saturated Ca(OH)_2_ solution at 90 °C with a 1-min interval.

### Heat treatment

The specimen was placed in an electric furnace (Ajeon), raised to 500 °C at a heating rate of 10 °C/min, and maintained for 2 h to achieve structural stabilization and impurity removal.

### Simulated body fluid immersion test

In order to evaluate the bioactivity, the specimen was immersed in SBF in whose pH and inorganic ion concentration were adjusted to a concentration similar to that of human plasma, and the material deposited on the surface was investigated. All specimens were autoclaved at 120 °C for 20 min, individually placed in conical tubes containing 10 ml of SBF, and then incubated for 1 or 2 days in a 37 °C incubator with a 5% CO_2_ atmosphere. The SBF was prepared by adding 0.185 g/L of calcium chloride dihydrate (CaCl_2_H_4_O_2_), 0.09767 g/L of magnesium sulfate (MgSO_4_), and 0.350 g/L of sodium hydrogen carbonate (NaHCO_3_) to a Hanks solution (H2387; Sigma-Aldrich). Its pH was adjusted to 7.4 using 1 N hydrogen chloride (HCl) solution.

### Cytotoxicity test

Cytotoxicity tests were performed using mouse-derived osteoblasts cell line MC3T3-E1. The culture solution was prepared by adding fetal bovine serum (FBS; Gibco) and antibiotics 500 unit/ml (Gibco) to *α*-MEM (Gibco). Cell culture was performed in a 37 °C incubator with a 5% CO_2_ atmosphere.

For the cytotoxicity test, UV sterilized specimens of each group according to ISO 10993-5:1999 were eluted in the media for 72 h, and then, WST-8 assay (Enzo cell counting kit-8; Enzo Life Sciences) test, one of the MTT assays, was performed. MC3T3-E1 cells were seeded into 48 well plates at a density of 1 × 10^4^ per well, and its medium was replaced with the eluted medium after 3 h. Cell culture was performed in a 37 °C incubator with a 5% CO_2_ atmosphere for 2, 4, or 7 days, and its medium was replaced with the eluted medium every 2 days. The 500 μl of the WST-8 assay reagent was added to each well and incubated for 90 min in a 37 °C incubator with a 5% CO_2_ atmosphere. Subsequently, 200 μl of the solution was transferred to each well of a 96-well plate and the absorbance at 450 nm was measured with an ELISA microplate reader (Model Spectra MAX PLUS; Molecular Devices).

### Animal implantation and removal torque test

This study was conducted in compliance with the principles of the Helsinki Declaration, and ethical clearance was obtained from the Institutional Animal Care and Use Committee of the Chonbuk National University Laboratory Animal Center (approval number: CBU 2012-0027).

A total of three 8-week-old New Zealand white rabbit males were used as animals for the mini-screw insertion experiment. The experiment was carried out after basic breeding for 1 week in an animal breeding room where the temperature and humidity are kept constant. Each rabbit was injected intramuscularly with 0.7 ml/kg of ketamine and 0.2 ml/kg of xylazine HCl to induce general anesthesia, followed by additional local anesthesia of the surgical site with 2% lidocaine added with epinephrine (1:100,000). Both legs of each rabbit were shaved, washed, and decontaminated with 10% povidone-iodine scrub. After exposing the flat surface of the tibia diaphysis with an incision, three mini-screws from the UT, AH, and APH groups were placed vertically on each tibia, approximately 10 mm apart. The self-drilling (without a pilot hole) insertion technique was used as the mini-screw placement method, and a total of 18 mini-screws were implanted in this study. After confirming the mini-screws were stable, the mucoperiosteum and muscles were sutured using absorbable sutures and surgical wounds were disinfected. To prevent infection after surgery, 4 ml of aminoglycoside antibiotic was intramuscularly injected for three days, and the wound was disinfected with 10% povidone-iodine scrub every day. Animals were sacrificed using isoflurane 4 weeks after surgery. Immediately after sacrifice, removal torque tests were performed using a digital torque gauge (9810P; Aikoh Engineering) with a precision of 0.1 N∙cm (*n* = 5 for each group).

### Surface analysis

The mini-screw was immediately immersed in physiological saline for about 1 h after removal, washed by shaking in distilled water 5 times, dried, and then coated with osmium. The morphological microstructure of the surface of the removed mini-screws was observed with a Field Emission Scanning Electron Microscope (FE-SEM, S800; Hitachi), and the change in the concentration of elements in the coating layer was observed with an X-ray spectrum (EDS; Bruker).

## Results

**Fig. 1:**
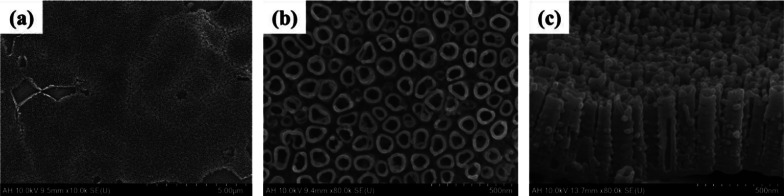
Field emission scanning electron microscope (FE-SEM) images of a Ti–6Al–4V ELI alloy plate of the AH group. **a** Top view with 10 k × magnification, **b** Top view with 80 k × magnification, **c** Cross-sectional view with 80 k × magnification

The FE-SEM images in Fig. 1 show the surface of a Ti–6Al–4V ELI alloy plate specimen of the AH group heat-treated at 500 °C after applying a voltage of 20 V for 60 min in a glycerol electrolyte solution containing 1.4 wt% NH_4_F and 20 wt% H_2_O. After the anodization treatment, nanotubes were formed on the surface of the AH group specimen (Fig. 1a, b). The nanotubes were fully self-aligned and had a dense structure in which the outer walls were bonded to each other (Fig. 1c).

**Fig. 2:**
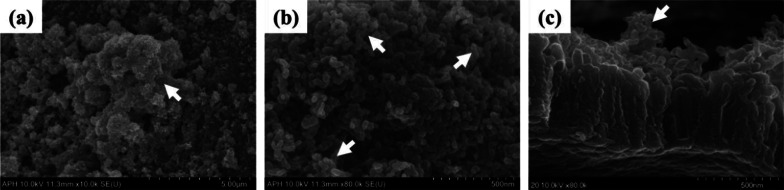
FE-SEM images of the surface of a Ti–6Al–4V ELI alloy plate specimen of the APH group. **a** Top view with 10 k × magnification, **b** Top view with 80 k × magnification, **c** Cross-sectional view with 80 k × magnification. White arrows indicate where precipitates are located

Figure 2 shows the surface of a Ti–6Al–4V ELI alloy plate specimen of the APH group made by anodizing, immersing 20 times in 0.05 M NaH_2_PO_4_ solution, and saturated Ca(OH)_2_ solutions at 90 °C with 1-min interval, and then heat treatment at 500 °C. Figure 2a, b are top view images for observing the specimen surface. In the 50 k × magnified image of Fig. 2b, it was observed that fine granular calcium phosphate precipitates formed clusters covering the nanotube layer. Figure 2c is an image of the cross section observed after the specimen was bent and the coating layer was fractured. It was observed that calcium phosphate precipitates locally penetrated and bonded to the inlet of the nanotubes.

The bioactivity was evaluated by immersing the specimens of all groups in SBF similar to human plasma and examining the material deposited on the surface. Table 1 shows the results of EDS analysis. Calcium (Ca) and phosphorus (P) were not detected on the surface of the specimen in the UT group, but Ca was detected on their surfaces in the AH and APH groups. In particular, compared with the AH group, the Ca concentration was higher on the surface of the specimen in the APH group, and P was also detected.

**Table 1:** Analysis results of Ca and P concentrations on the surfaces of Ti–6Al–4V ELI alloy plate specimens of the UT, AH, and APH groups immersed in SBF for 1 day

	UT	AH	APH
Ca (wt%)	–	0.06	27.34
P (wt%)	–	–	12.45
Ca/P (at%)	–	–	1.70

**Fig. 3:**
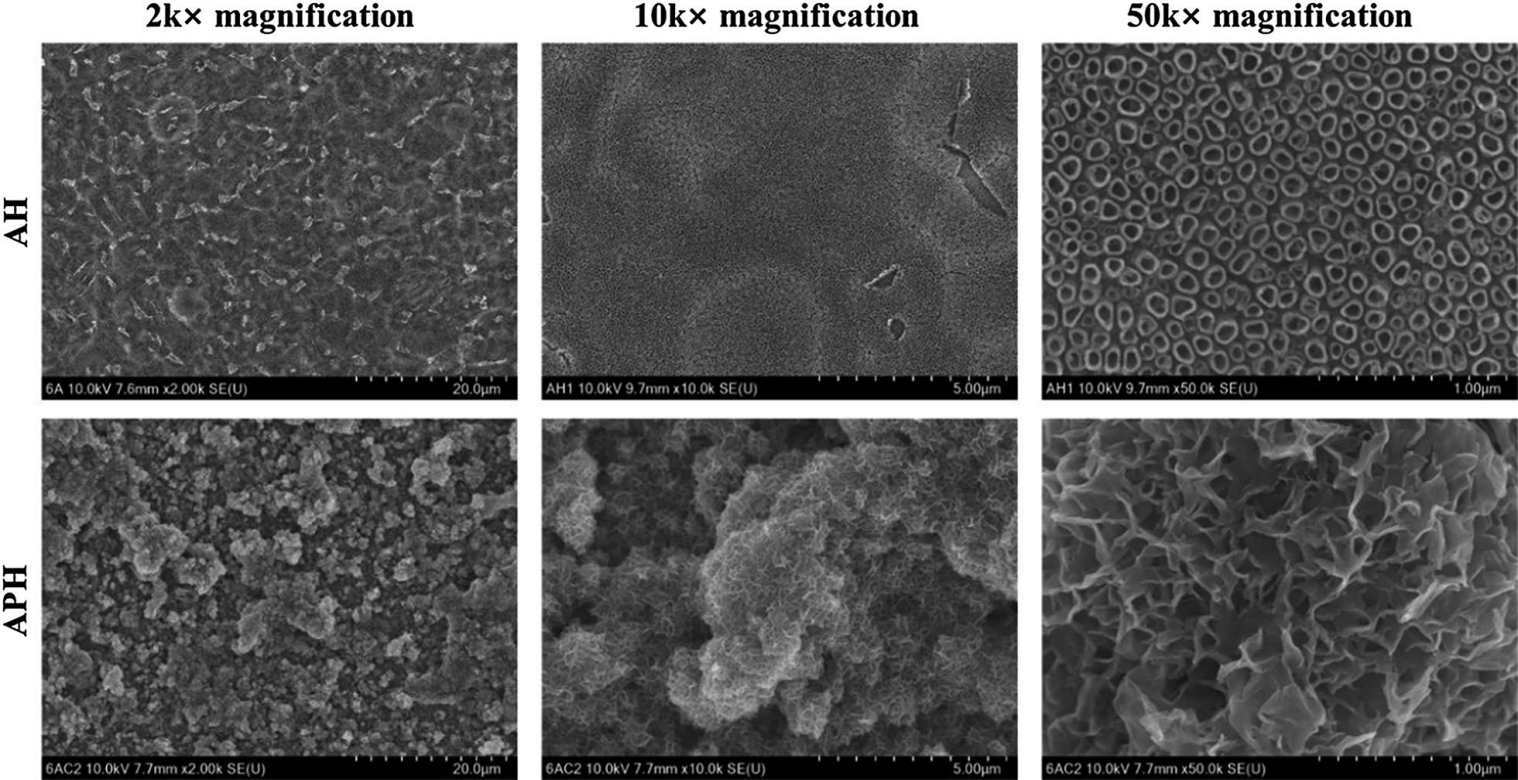
FE-SEM images of the surface of a Ti–6Al–4V ELI alloy plate specimen of the APH group immersed in SBF for 1 days

Figure 3 shows the FE-SEM images of the surfaces of the specimens in the AH and APH groups, and protrusions were also observed in the 50 × magnified image of the surface of the APH group in aspect of the structure of the surface.

**Fig. 4:**
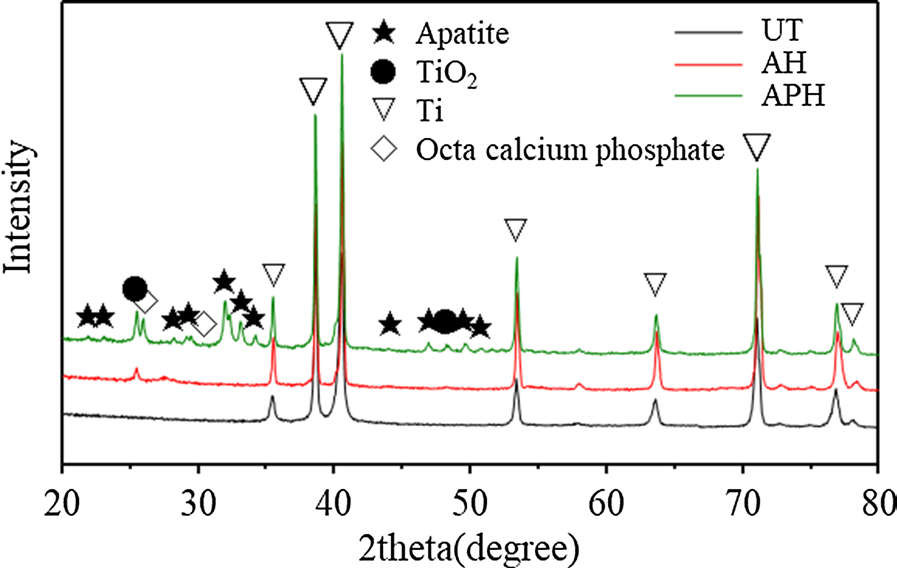
X-ray diffraction patterns of Ti–6Al–4V ELI alloy plate specimens for the UT, AH, and APH groups

Figure 4 indicates the results of X-ray diffraction analysis of the specimens for the UT, AH, and APH groups. In the UT group, only Ti peak was observed, and in the AH group, TiO_2_ peak was observed along with Ti peak. However, in the APH group, in addition to these peaks, the peaks of octacalcium phosphate and HA were observed.

**Fig. 5:**
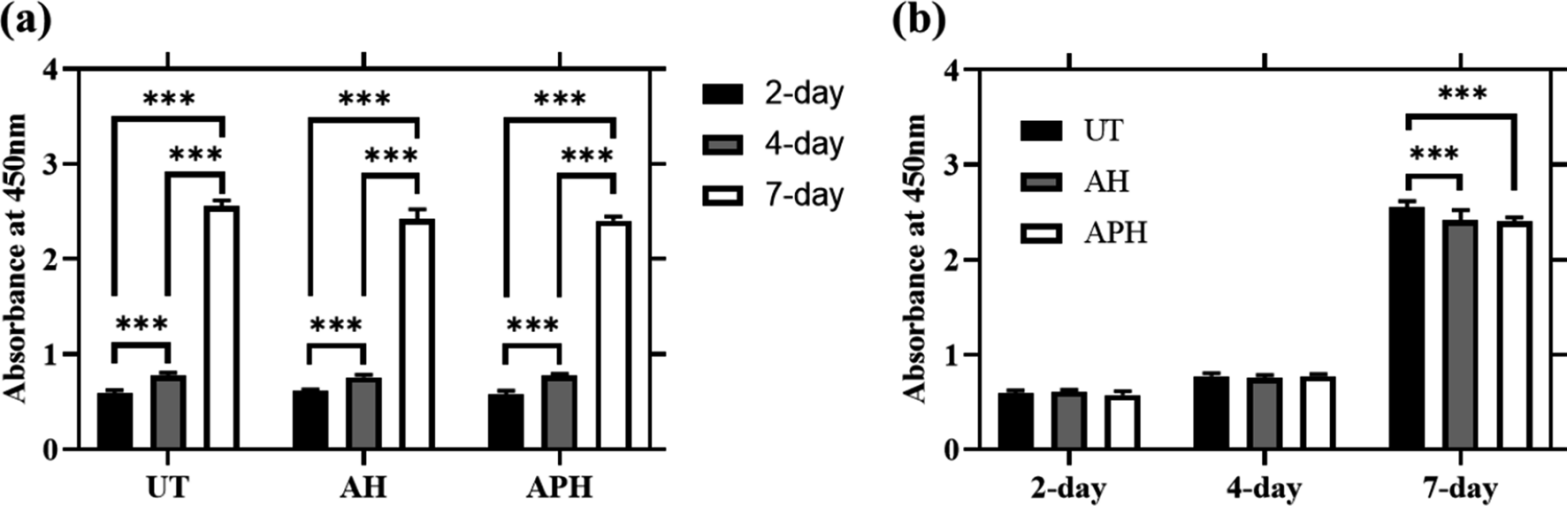
Cell proliferation behavior was measured by WST-8 assay after incubating MC3T3-E1 on specimens for the UT, AH, and APH groups for 1, 4, and 7 days. **a** Comparison between days, **b** Comparison between groups. The data were statistically analyzed by two-way analysis of variance (ANOVA) followed by Tukey’s multiple comparisons test. The results are presented as the means ± standard deviation for *n* = 18 (*** denotes a *p* value of < 0.005)

The proliferation of MC3T3-E1 cells on specimens of the UT, AH, and APH groups was assessed by WST-8 assay analysis after 2, 4, and 7 days of culture. As shown in Fig. 5, MC3T3-E1 cell proliferation occurred significantly with the elapse of days in all groups. On 2- and 4-day, the cell proliferation occurred without significant differences between groups, but on 7-day, the UT group showed significantly higher cell density than other groups. In the UT group, the absorbance was 0.59 ± 0.03 on 2-day, 0.78 ± 0.03 on 4-day, and 2.56 ± 0.06 on 7-day; in the AH group, it was 0.61 ± 0.02 on 2-day, 0.76 ± 0.03 on 4-day, and 2.42 ± 0.11 on 7-day; and in the APH group, it was 0.58 ± 0.04 on 2-day, 0.78 ± 0.02 on 4-day, and 2.40 ± 0.04 on 7-day.

In the same manner as the Ti–6Al–4V ELI plate test, mini-screws were also classified into UT, AH, and APH groups, and the surface was confirmed through FE-SEM after surface treatment suitable for each group. Additional file 1: Figure S1 shows the surface of a mini-screw with the same surface treatment as the Ti–6Al–4V ELI plate specimen of the AH group. The surface was heat-treated at 500 °C after applying a voltage of 20 V for 60 min in a glycerol electrolyte containing 1.4 wt% NH_4_F and 20 wt% H_2_O. Similar to the surface treatment results of the plate specimen for the AH group, it was confirmed that nanotubes having a fully self-aligned dense structure were formed on the surface of the mini-screw (Additional file 1: Figure S1b and c).

**Fig. 6:**
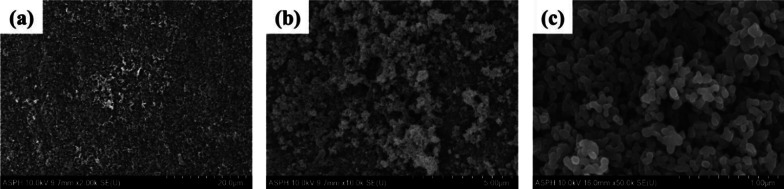
FE-SEM images of the surface of a Ti–6Al–4V ELI alloy mini-screw of the APH group. **a** 2 k × magnification, **b** 10 k × magnification, **c** 50 k × magnification

Figure 6 shows the surface of the mini-screw for the APH group. Its surface was formed by anodizing, immersing 20 times in 0.05 M NaH_2_PO_4_ solution and saturated Ca(OH)_2_ solutions at 90 °C with 1 min intervals, and then heat-treating at 500 °C. Similar to the surface treatment results of plate specimen for the APH group, it was observed that fine granular calcium phosphate precipitates formed clusters covering the nanotube layer on the surface of the mini-screw (Fig. 6b, c).

**Fig. 7:**
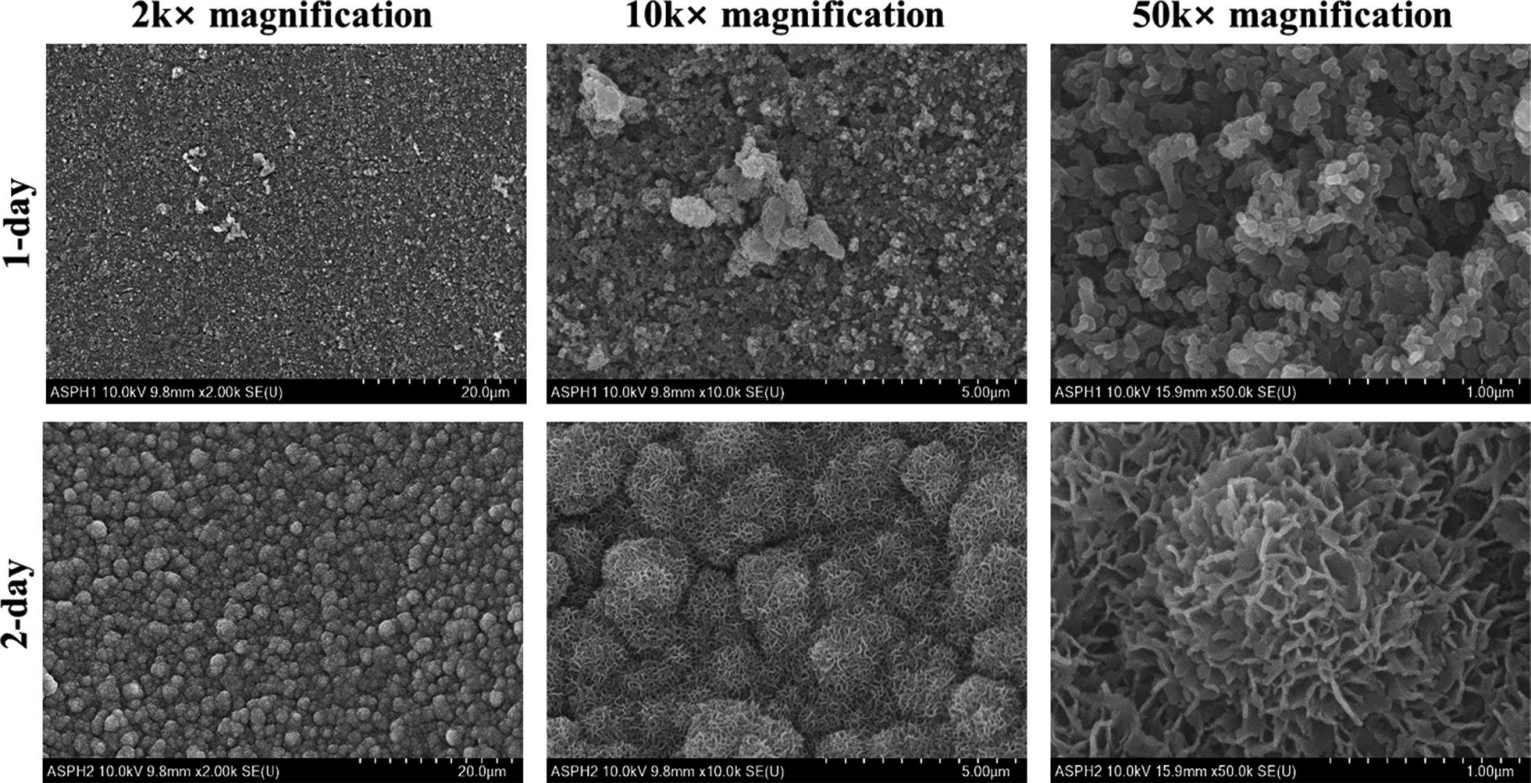
FE-SEM images of the surface of a Ti–6Al–4V ELI alloy mini-screw of the APH group immersed in SBF for 1 and 2 days

Figure 7 shows the surface of the mini-screws of the APH group immersed in SBF for 1 and 2 days. There was no significant difference in the surface of the mini-screws for the APH group immersed in SBF for 1 day, but after 2 days of immersion, the densely structured protrusions were observed.

To measure the removal torque and degree of osseointegration, mini-screws from UT, AH, and APH groups were placed in rabbit tibias, and extractions were performed 4 weeks later.

**Fig. 8:**
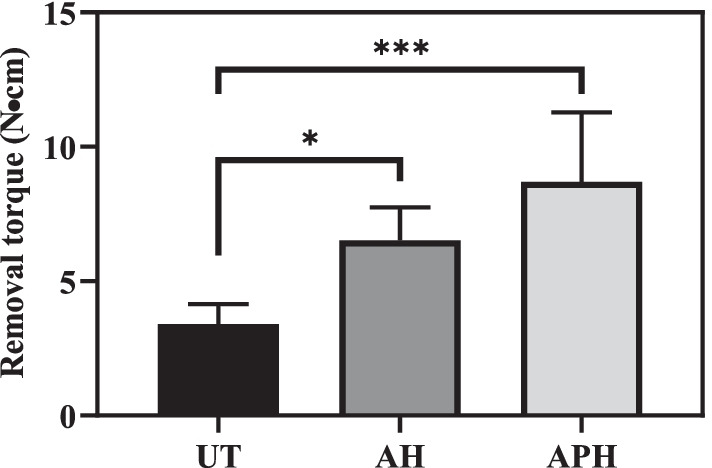
Mini-screw removal torque values were measured 4weks after implantation into rabbit tibias. The values were statistically analyzed by one-way ANOVA followed by Tukey’s multiple comparisons test. The results are presented as the means ± standard deviation for *n* = 5 (* denotes a *p* value of < 0.05; *** denotes a *p* value of < 0.005)

Figure 8 shows the removal torque values measured 4 weeks after implantation into rabbit tibias. The values were 3.40 ± 0.74, 6.52 ± 1.22, and 8.70 ± 2.59 N·cm in the UT, AH, and the APH groups, respectively. The values of the AH and APH groups were significantly higher than that of the UT group, and the APH group had the highest value.

**Fig. 9:**
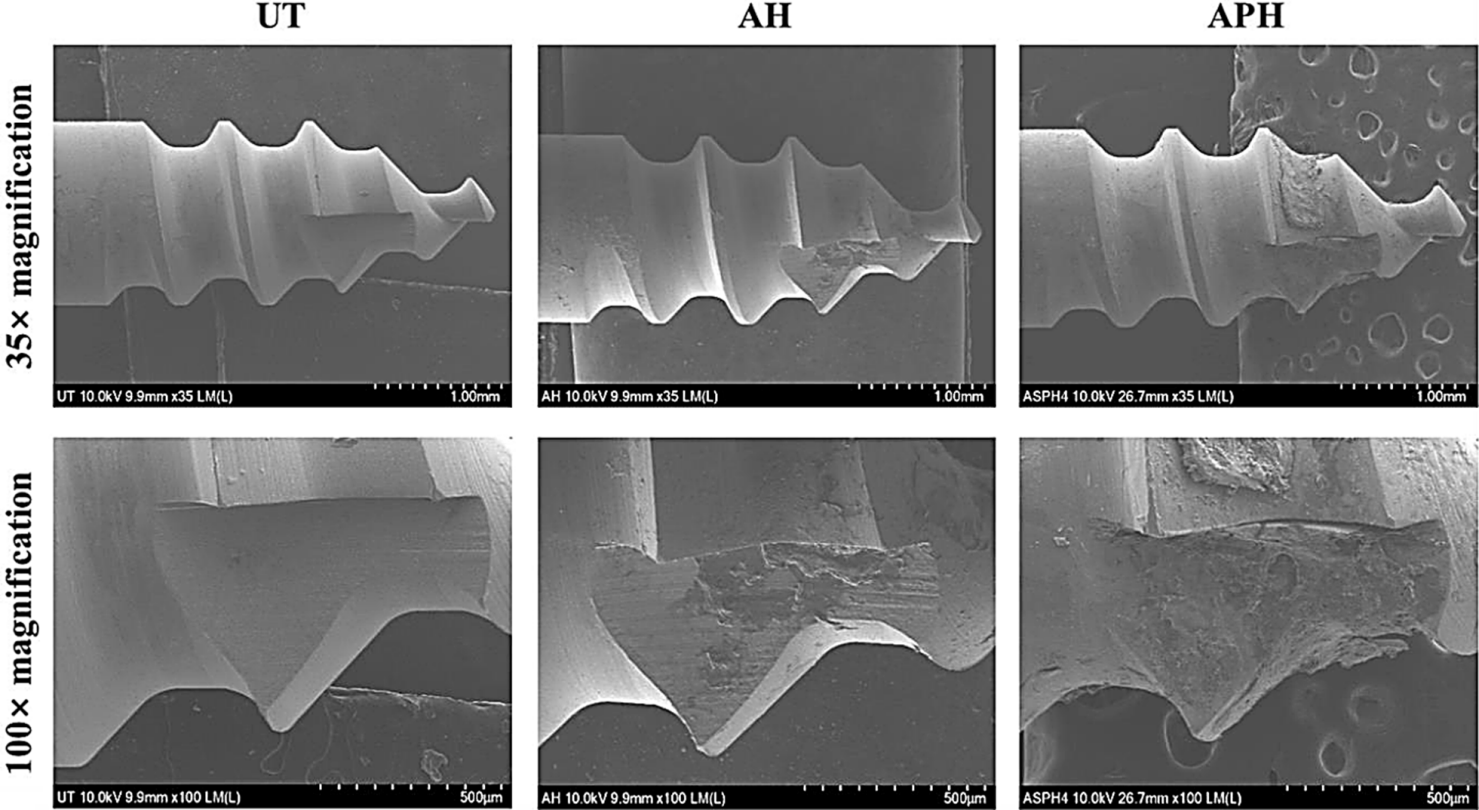
FE-SEM images of the UT, AH, and APH group mini-screws removed 4 weeks after implantation into rabbit tibias

Figure 9 shows FE-SEM images of the mini-screw surfaces of the UT, AH, and APH groups extracted 4 weeks after implantation into rabbit tibias. In all of the five mini-screws of the UT group, fracture occurred at the interface without attachment of osseous tissue, so that uniform directional machining traces generated during CNC machining could be observed. On the other hand, in all the mini-screws of the AH and APH groups, not only interfacial fractures but also cohesive fractures caused by the local attachment of osseous tissue to their surface occurred. Cohesive fractures were observed on a larger surface in the APH group.

**Fig. 10:**
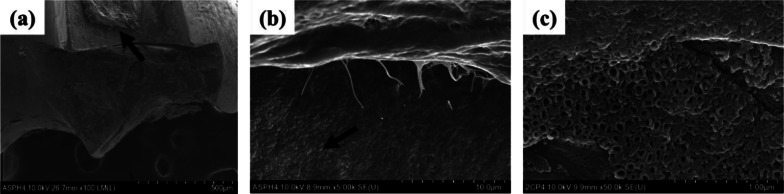
Surface morphology of the APH group mini-screw removed 4 weeks after implantation into rabbit tibias. **a** 100 × magnification, **b** 5 k × magnification, **c** 50 k × magnification. The arrows in **a** and **b** indicate the locations where **b** and **c** images were taken, respectively

Figure 10 shows the surface morphology of the APH group mini-screw extracted 4 weeks after implantation into rabbit tibias. In the upper part of Fig. 10b, cohesive fractures with osseous tissue attached occurred, and interfacial fractures were shown in the lower part. Figure 10c is an image of the interfacial fracture at 50 k magnification, and it can be confirmed that the fracture occurred at the interface with the nanotube-structured TiO_2_ layer.

## Discussion

Metals must have good corrosion resistance, high mechanical properties, and biocompatibility for use in the oral environment. Among various types of titanium alloys, the Ti—6Al–4V ELI is known to have excellent corrosion resistance, mechanical properties, and biocompatibility as a representative *α* + *β* type titanium alloy containing *α* stabilizer element Al and β stabilizer element V [24, 25]. In particular, the TiO 2 layer formed on the surface of Ti and its alloys has an advantage in terms of corrosion resistance that prevents titanium from being corroded in the in vivo environment but also has a disadvantage in that it is difficult to cause osseointegration [26, 27]. Therefore, this paper proposes a procedure to sequentially treat the surface of Ti–6Al–4V ELI alloy mini-screws through anodization, cyclic pre-calcification, and heat treatment as a method for promoting osseointegration.

A noteworthy feature in this paper is that Ca and P were detected in the result of EDS analysis after immersing the specimen of the APH group in SBF similar to human plasma for 1 day (Table 1). Calcium phosphate (CaPO_4_) materials in implantable devices have been reported to have several interesting properties [28, 29]: composition similarity with bone minerals, ability to form carbonate hydroxyapatite on their surface, ability to promote cellular function and expression leading to the formation of a strong bone-CaPO_4_ biomaterial interface, providing adequate scaffolds for bone formation, ability to bind and concentrate endogenous bone morphogenetic proteins. However, since histological verification was not performed in this paper, it is necessary to investigate whether these properties appear by performing the verification in future studies.

The other feature is that fluorine-containing hydrofluoric acid and ammonium fluoride were used in the process of pickling treatment to remove metal contaminants and clean the surface, and in the process of forming the nanotube structure layer to promote adhesion and proliferation of osteoblasts. As shown in Fig. 5b, the reason why the absorbance of the APH group is significantly lower than that of other groups is presumed to be due to the fluorine remaining in the nanotubes interfering with cell proliferation [30, 31]. However, it cannot be judged that residual fluorine unconditionally has a bad effect. This is because fluorine modification on the surface of titanium or titanium alloys has been demonstrated by previous studies to promote osteoblast formation and osseointegration [32–34]. Chen et al. [35] also showed that depositing fluorine on the titanium surface is a suitable strategy to simultaneously improve antimicrobial and osseointegration properties. As supporting these results, in the results of this paper, compared to the UT group, the APH group had more osseous tissue left on the surface of the mini-screw (Figs. 9, 10), and the removal torque was also significantly higher (Fig. 8). In light of these results, it seems that studies on the appropriate amount of fluoride and the duration of maintenance should be continued at all times.

## Conclusion

This paper was carried out to investigate whether inducing osseointegration by imparting bioactivity to the orthodontic mini-screw surface improves retention. Based on the results of this paper, the proposed orthodontic mini-screw is a Ti–6Al–4V ELI alloy mini-screw that was made by anodizing, immersing 20 times in 0.05 M NaH_2_PO_4_ solution and saturated Ca(OH)_2_ solutions at 90 °C with 1 min intervals, and then heat treatment at 500 °C. The features of this mini-screw are as follows:

1. Its surface is formed with a layer of nanotubes having a dense structure in the form of fully self-aligning.
2. On its surface, fine granular calcium phosphate precipitates of HA and octacalcium phosphate are formed as clusters.
3. Through SBF immersing, Ca and P concentrations, which bind and concentrate endogenous bone morphogenetic proteins, increased on the surface.
4. Compared to the UT group, its removal torque is significantly higher.

## Supplementary Information

**Additional file 1**. Figure S1.

## Data Availability

Not applicable.

## Abbreviations

Ti: Titanium
HA: Hydroxyapatite
SBF: Simulated body fluid
UT: Untreated
AH: Anodized and heat-treated
APH: Anodized, pre-calcified, and heat-treated
CNC: Computer numerical control
FBS: Fetal bovine serum
FE-SEM: Field emission scanning electron microscope
