# Supplementary material for: Improvement of osseointegration of Ti–6Al–4V ELI alloy orthodontic mini-screws through anodization, cyclic pre-calcification, and heat treatments
Source: Prog Orthod. 2022 Apr 4;23:11. doi: 10.1186/s40510-022-00405-8 (PMC8977256; doi:10.1186/s40510-022-00405-8)

Figure S1. FE-SEM images of a Ti-6Al-4V ELI alloy mini-screw of the AH group. (a) Top view with 10k × magnification, (b) Top view with 100k × magnification, (c) Cross-sectional view with 100k × magnification.
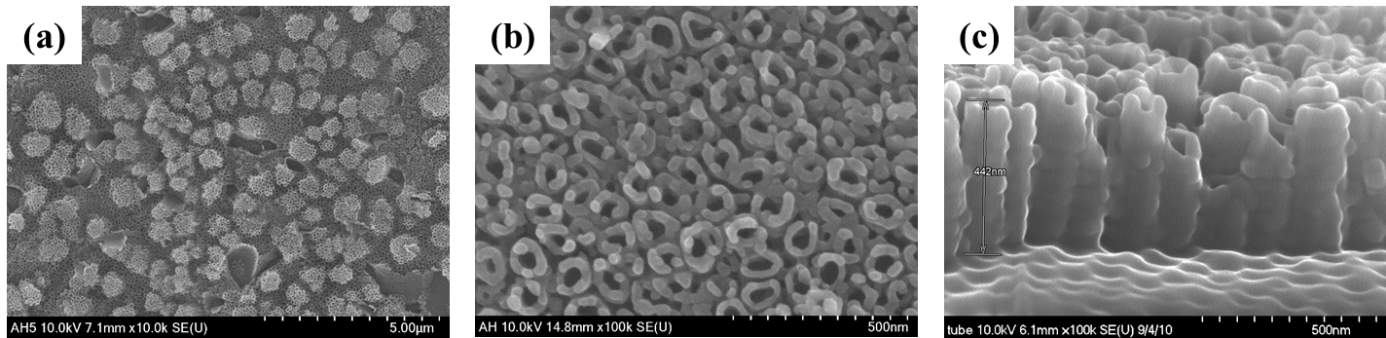

Supplement: Supplementary file 1 — Additional file 1. Figure S1. [file 40510_2022_405_MOESM1_ESM.docx]
